# Effects of parathyroidectomy on plasma PTH fragments and heart rate variability in stage 5 chronic kidney disease patients

**DOI:** 10.1080/0886022X.2021.1931318

**Published:** 2021-05-27

**Authors:** Huimin Chen, Wenkai Ren, Zhanhui Gao, Ming Zeng, Shaowen Tang, Fangyan Xu, Yaoyu Huang, Lina Zhang, Ying Cui, Guang Yang, Hanyang Qian, Wenbin Zhou, Chun Ouyang, Xueyan Gao, Jing Zhang, Yujie Xiao, Baiqiao Zhao, Jing Wang, Anning Bian, Fan Li, Huiting Wan, Wei Gao, Xiaoyun Wang, Changying Xing, Xiaoming Zha, Ningning Wang

**Affiliations:** a Department of Nephrology, The First Affiliated Hospital of Nanjing Medical University, Jiangsu Province Hospital, Nanjing, China; b Department of Nephrology, Taizhou People’s Hospital, Taizhou, China; c Department of Nephrology, BenQ Medical Center, The Affiliated BenQ Hospital of Nanjing Medical University, Nanjing, China; d Department of Epidemiology and Biostatistics, School of Public Health, Nanjing Medical University, Nanjing, China; e Department of Nephrology, Henan Provincial Key Laboratory of Kidney Disease and Immunology, Henan Provincial People’s Hospital, People’s Hospital of Zhengzhou University, Zhengzhou, China; f Department of Nephrology, Northern Jiangsu People’s Hospital Affiliated to Yangzhou University, Yangzhou, China; g Department of General Surgery, The First Affiliated Hospital of Nanjing Medical University, Jiangsu Province Hospital, Nanjing, China; h Department of Nephrology, Liyang Branch, Jiangsu Province Hospital, Liyang People’s Hospital, Liyang, China; i Department of General Medicine, Geriatric Hospital of Nanjing Medical University, Nanjing, China; j National Health Commission Key Laboratory of Antibody Techniques, School of Basic Medical Sciences, Nanjing Medical University, Nanjing, China

**Keywords:** Chronic kidney disease, (1-84) PTH, (7-84) PTH, heart rate variability, secondary hyperparathyroidism, parathyroidectomy

## Abstract

**Introduction:**

Circulating intact parathyroid hormone (iPTH) levels include full-length (1-84) PTH and long C-PTH fragments, but primarily (7-84) PTH, which have been reported to have antagonistic effects on the bones and kidneys. However, their effects on the cardiovascular system remain unclear. In this study, the relationships between the plasma PTH fragments levels and heart rate variability (HRV) in stage 5 chronic kidney disease (CKD5) patients are explored. Furthermore, the effects of parathyroidectomy (PTX) on the above indices are investigated.

**Methods:**

In this cross-sectional study, 164 healthy controls and 354 CKD5 patients, including 208 secondary hyperparathyroidism (SHPT) subgroup with PTX, were enrolled. Circulating (7-84) PTH levels were calculated by subtracting plasma (1-84) PTH levels from iPTH levels. The HRV parameters were measured using a 24-hour Holter.

**Results:**

The baseline levels of plasma iPTH, (1-84) PTH, and (7-84) PTH in the CKD5 patients were 930.40 (160.65, 1792.50) pg/mL, 448.60 (99.62, 850.45) pg/mL, and 468.20 (54.22, 922.55) pg/mL, respectively. In the CKD5 patients, plasma (1-84) PTH levels were independently correlated with the standard deviation of the normal-to-normal R-R intervals (SDNN) and the standard deviation of the five-minute average of the normal R-R intervals (SDANN). With a median follow up time of 6.50 months after PTX in the SHPT patients (*n* = 30), improved SDNN and SDANN markers were related with decreased (1-84) PTH levels. Furthermore, an improved SDNN was related with decreased (7-84) PTH levels.

**Conclusions:**

The CKD5 patients’ baseline (1-84) PTH levels were correlated with the SDNN and SDANN. After PTX, an improved SDNN was related with decreased (1-84) PTH and (7-84) PTH levels, while improved SDANN was related with decreased (1-84) PTH levels. No antagonistic effects of (1-84) PTH and (7-84) PTH on HRV were found in the CKD5 patients.

## Introduction

Cardiovascular disease (CVD) is the leading causes of death in patients with end-stage kidney disease (ESKD). The risk of cardiovascular disease in patients with chronic kidney disease (CKD) is 5–10 times higher than that in the general population [1]. The United States Renal Data System 2019 report showed that the incidence of CVD in CKD patients over 65 years old was over half of the cohort [2]. Patients in stage 5 CKD (CKD5) with severe secondary hyperparathyroidism (SHPT) are at increased risk of cardiovascular disease and death due to increased synthesis and secretion of parathyroid hormone (PTH) [3–5]. However, a successful parathyroidectomy (PTX) can significantly reduce the serum PTH levels and risks for all-cause and cardiovascular mortality [6].

Cardiac sympathetic overdrive and decreased vagal control appear during CKD 4 and 5 leading to arrhythmias, which are the most important causes of sudden cardiac death in CKD, especially in hemodialysis patients [7]. Heart rate variability (HRV) is a noninvasive measurement to evaluate autonomic dysfunction that reflects beat-to-beat variability in the heart rate. A decrease in the HRV has an independent predictive value for the occurrence of various CVDs, especially ventricular arrhythmias and sudden cardiac death [8–10]. Our previous study proved that HRV indices decreased significantly in CKD5 patients, especially in patients with severe SHPT, while PTX could improve the reduced HRV parameters [11,12].

Circulating PTH levels are currently measured using the second generation intact PTH (iPTH) assays that detect different PTH fragments, including full-length (1-84) PTH and long C-PTH fragments, primarily (7-84) PTH [13]. The latter has been demonstrated to have antagonistic effects against (1-84) PTH for the bones and kidneys [14]. The third generation PTH assays have been proved to recognize (1-84) PTH specifically [14], which have not been widely used in clinical practice.

Few studies have reported the effects of different PTH fragments on the potential diagnosis and prognosis of CVD [15], and whether the effects of (1-84) PTH and (7-84) PTH on the cardiovascular system of CKD5 patients are different remains unclear. This study is aims to explore the relationships between the different PTH fragments and the HRV parameters in CKD5 patients. Furthermore, the effects of PTX on the above indices will be investigated using a follow-up of the severe SHPT patients. This research provides new insights for the pathogenesis and potential therapeutic targets for cardiovascular diseases in patients with CKD and severe SHPT.

## Materials and methods

### Study population

In this study, 354 stage 5 CKD patients were enrolled from October 2013 to October 2018 from the Department of Nephrology, the First Affiliated Hospital of Nanjing Medical University and the BenQ Hospital of Nanjing Medical University. The inclusion criteria of the patients included: (1) ages 18–75 years; (2) estimated glomerular filtration rate (eGFR)<15 mL/min/1.73 m^2^; and (3) hemodialysis patients dialyzed three times a week for four hours each time using bicarbonate dialysate.

Exclusion criteria of the patients included [13,16]: (1) undergoing maintenance dialysis between 0 and 3 months; (2) persistent SHPT patients who underwent PTX before enrollment and required the second PTX due to residual hyper-functional parathyroid tissue; (3) had a history of kidney transplantation; (4) a fasting blood glucose on the day of evaluation ≥200 mg/dL; (5) the presence of fever, infection or pregnant women; (6) severe congenital heart disease, atrial fibrillation or flutter, high-grade atrioventricular block, permanent pacemaker implantation; (7) severe hepatic disease, chronic obstructive lung disease, malignant tumors, or severe mental disorders; (8) episodes of acute myocardial infarction, stroke, or a major surgical procedure within the past two months; and (9) the use of immunosuppressive drugs, calcitonin, or bisphosphonates.

In 354 of the CKD5 patients, 208 had severe SHPT and received total PTX with forearm auto-transplantation. The surgical indications were the same as the references [13,16,17]. Successful PTX is defined as blood iPTH levels < 50 pg/mL within one week after surgery [18]. Thirty PTX patients were followed up after three months to explore the effects of PTX on the HRV parameters, for whom blood tests and a 24-h Holter were conducted before and after the operation.

A total of 164 healthy volunteers aged between 18 and 75 years old in different centers were enrolled as the control group, and women who were pregnant or lactating were excluded.

The basic medical information of the CKD5 patients, such as demographics, dialysis mode, medical history, causes of CKD, and baseline laboratory tests, were recorded at the time of enrollment. Additionally, the use of antihypertensive drugs in the patients was recorded, including angiotensin converting enzyme inhibitor (ACEI), angiotensin receptor blocker (ARB), β-adrenergic receptor blocker, and calcium channel blockers (CCB). Healthy people took questionnaires to obtain the personal information.

All of patients and controls participating in the study gave written informed consent. The study protocol was approved by the Research Ethics Committee of the First Affiliated Hospital of Nanjing Medical University, Nanjing, China (2011-SR-072.A1, 2018-SR-207).

### Determinations of the blood parameters and ambulatory heart rate monitoring

Fasting venous blood in the morning was examined for routine blood tests, blood biochemistry, and different PTH fragments. The HRV examinations in hemodialysis patients were conducted during the interval between two dialysis sessions, and the above examinations were completed within two weeks prior to the PTX. The above indices were repeated in the PTX subgroup when they were followed up.

Elecsys^®^ iPTH and Elecsys^®^ (1-84) PTH kits (Cobas, Roche Diagnostics, Mannheim, Germany) were measured using the electrochemiluminescence immunoassay (ECLIA) method. The circulating (7-84) PTH levels were calculated by subtracting the plasma (1-84) PTH levels from the plasma iPTH levels [19].

The HRV indices were obtained using 24-h Holter examinations [12]. The HRV parameters included: (1) the mean heart rate (MHR); (2) the time domain indices: mean normal-to-normal R-R intervals (mean NN); standard deviation of the normal-to-normal R-R intervals (SDNN); standard deviation of the five-minute average of the normal R-R intervals (SDANN); the root-mean square of differences between the adjacent normal R-R intervals (rMSSD); the percentage of the adjacent NN intervals differing by more than 50 milliseconds (pNN50); and (3) the frequency domain indices: very-low frequency (VLF); low-frequency (LF); high-frequency (HF); and the LF/HF.

### Statistical analysis

The categorical variables were presented as numbers and proportions, and the continuous variables were presented as means ± standard deviations (SD) or medians (interquartile range). The data of the significant skewness distribution were transformed using the natural logarithm. Differences between the groups were compared using independent samples, a one-way ANOVA, or the Wilcoxon rank-sum test for continuous variables and the chi-square test or exact probability test for the categorical variables. To adjust for confounding factors, a single-variable regression analysis was first conducted to select the statistically significant variables (*p* < .1) as independent variables. Then, multiple stepwise regression models were used to identify the HRV factors. A paired sample *t*-test was used to assess the differences between the values recorded before and after the PTX. A *p* < .05 was considered statistically significant. All of the statistical analyses were performed using the statistical package for the social sciences (SPSS), version 25.0 (SPSS Inc., Chicago, IL, USA).

## Results

### Characteristics of the study population

A total of 354 CKD5 patients were enrolled, with an average age of 49.57 ± 11.76 years old, including 192 males and 162 females. Compared with controls, the CKD5 patients had higher blood pressure (BP), creatinine, alkaline phosphatase (ALP), triglycerides (TG), calcium (Ca), phosphorus (P), and lower hemoglobin, albumin, and total cholesterol (TC) levels (*p* < .001). Among the CKD5 group, 56.50% patients had taken active vitamin D, 54.80% had phosphorus binder, and 13.84% had taken cinacalcet (Table 1). The plasma iPTH, (1-84) PTH and (7-84) PTH levels in the controls were 31.47 (22.48, 41.03) pg/mL, 23.05 (16.81, 33.61) pg/mL, and 6.29 (3.79, 9.98) pg/mL, respectively. While in the stage 5 CKD patients, the baseline levels of plasma iPTH, (1-84) PTH, and (7-84) PTH were 930.40 (160.65, 1792.50) pg/mL, 448.60 (99.62, 850.45) pg/mL, and 468.20 (54.22, 922.55) pg/mL, respectively. The value of the (1-84) PTH/iPTH ratio was 0.79 in the controls and obviously reduced in the CKD patients at 0.58.

**Table 1.** Demographics, clinical characteristics and laboratory results in controls and stage 5 CKD patients.

Variables	Controls (*n* = 164)	Stage 5 CKD Patients (*n* = 354)	*p* Value^a^	Stage 5 CKD Patients						
				iPT*H* ≤ 50 (*n* = 30)	50 < iPT*H* ≤ 150 (*n* = 53)	150 < iPT*H* ≤ 300 (*n* = 41)	300 < iPT*H* ≤ 800 (*n* = 37)	800 < iPT*H* ≤ 1500 (*n* = 80)	iPT*H* > 1500 (*n* = 113)	*p* value^b^ (ANOVA)
Demographics										
Age (years)	42.99 ± 14.46	49.57 ± 11.76	<.001	53.27 ± 12.29	50.55 ± 13.14	52.63 ± 12.35	51.14 ± 10.37	48.96 ± 10.87	46.95 ± 11.34	.026
Male/Female	60/104	192/162	<.001	19/11	27/26	27/14	18/19	39/41	62/51	.426
BMI (kg/m^2^)	22.76 ± 3.05	22.34 ± 3.32	.172	22.59 ± 4.17	21.92 ± 3.36	22.14 ± 3.34	23.28 ± 3.13	23.08 ± 3.05	21.75 ± 3.16	.057
SBP (mmHg)	119.28 ± 14.65	142.37 ± 23.66	<.001	153.77 ± 18.54	149.83 ± 22.78	151.32 ± 31.91	141.89 ± 25.87	135.77 ± 20.54	137.37 ± 20.25	<.001
DBP (mmHg)	75.43 ± 10.27	85.34 ± 13.59	<.001	85.97 ± 16.99	84.74 ± 12.48	87.32 ± 13.21	85.32 ± 15.83	82.70 ± 11.78	86.60 ± 13.61	.417
Dialysis mode, *n* (%)										
Predialysis	0 (0.00)	30 (8.47)	/	5 (16.67)	8 (15.09)	10 (24.39)	5 (13.51)	2 (2.50)	0 (0.00)	/
Hemodialysis	0 (0.00)	281 (79.38)	/	22 (73.33)	37 (69.81)	23 (56.10)	27 (72.97)	66 (82.50)	106 (93.81)	<.001
Peritoneal dialysis	0 (0.00)	44 (12.43)	/	3 (10.00)	8 (15.09)	8 (19.51)	5 (13.51)	12 (15.00)	8 (7.08)	.317
*Dialysis vintage (months)*	0.00 (0.00–0.00)	66.50 (25.75–108.00)	/	18.00 (4.75–41.25)	16.00 (4.50–36.50)	24.00 (0.50–72.00)	60.00 (13.00–106.00)	84.00 (60.00–120.00)	96.00 (72.00–120.00)	<.001
Comorbidities, *n* (%)										
Diabetic mellitus	0 (0.00)	35 (9.89)	/	8 (26.67)	6 (11.32)	4 (9.76)	8 (21.62)	7 (8.75)	2 (1.77)	<0.001
Hypertension	15 (0.09)	285 (80.51)	<.001	27 (90.00)	47 (88.68)	33 (80.49)	31 (83.78)	56 (70.00)	91 (80.53)	0.079
Cause of ESKD, *n* (%)										
CGN	0 (0.00)	268 (75.71)	/	19 (63.33)	37 (69.81)	26 (63.41)	25 (67.57)	70 (87.50)	91 (80.53)	.008
DN	0 (0.00)	22 (6.21)	/	6 (20.00)	6 (11.32)	5 (12.20)	3 (8.11)	1 (1.25)	1 (0.89)	<.001
HN	0 (0.00)	39 (11.02)	/	14 (46.67)	10 (18.87)	11 (26.83)	3 (8.11)	1 (1.25)	0 (0.00)	/
PKD	0 (0.00)	12 (3.39)	/	1 (3.33)	2 (3.77)	2 (4.88)	4 (10.81)	1 (1.25)	2 (1.77)	.129
Other	0 (0.00)	51 (14.41)	/	4 (13.33)	8 (15.09)	6 (14.63)	8 (21.62)	7 (8.75)	18 (15.93)	.564
Medication history, *n* (%)										
CCB	0 (0.00)	192 (54.24)	/	22 (73.33)	36 (67.92)	30 (73.17)	15 (40.54)	33 (41.25)	56 (49.56)	<.001
ACEI/ARB	0 (0.00)	80 (22.60)	/	8 (26.67)	12 (22.64)	8 (19.51)	10 (27.03)	17 (21.25)	25 (22.12)	.961
β-receptor blocker	0 (0.00)	136 (38.42)	/	12 (40.00)	20 (37.74)	15 (36.59)	13 (35.14)	29 (36.25)	47 (41.59)	.970
Phosphate binder	0 (0.00)	194 (54.80)	/	22 (73.33)	30 (56.60)	19 (46.34)	22 (59.46)	41 (51.25)	60 (53.10)	.280
Active Vitamin D sterols	0 (0.00)	200 (56.50)	/	17 (56.67)	28 (52.83)	21 (51.22)	25 (67.57)	46 (57.50)	63 (55.75)	.753
Cinacalcet	0 (0.00)	49 (13.84)	/	0 (0.00)	1 (1.89)	4 (9.76)	7 (18.92)	19 (23.75)	18 (15.93)	/
Laboratory values										
Hemoglobin (g/L)	139.57 ± 15.39	100.34 ± 20.74	<.001	105.60 ± 16.35	99.06 ± 20.83	89.32 ± 18.00	99.41 ± 24.83	102.41 ± 18.99	102.38 ± 21.38	.007
Hematocrit (%)	41.75 ± 4.21	31.05 ± 6.40	<.001	32.37 ± 4.88	30.25 ± 6.36	27.39 ± 5.46	30.56 ± 7.53	31.72 ± 5.94	32.08 ± 6.56	.001
Glucose (mmol/L)	5.22 ± 0.75	5.04 ± 1.86	.109	6.34 ± 2.86	6.01 ± 2.28	5.58 ± 2.60	4.70 ± 0.97	4.77 ± 1.29	4.33 ± 0.93	<.001
Albumin (g/L)	47.88 ± 4.92	38.08 ± 4.93	<.001	37.99 ± 7.17	38.81 ± 5.59	36.75 ± 5.65	37.81 ± 4.30	38.59 ± 4.54	37.98 ± 3.96	.401
Creatinine (μmol/L)	68.09 ± 16.13	919.64 ± 305.19	<.001	832.66 ± 346.73	878.40 ± 329.08	997.33 ± 380.43	944.98 ± 380.71	952.10 ± 281.35	902.62 ± 227.48	.181
Urea (mmol/L)	5.17 ± 1.56	24.10 ± 8.19	<.001	24.49 ± 7.82	23.90 ± 8.37	27.20 ± 8.60	26.34 ± 10.44	22.97 ± 7.23	23.02 ± 7.58	.030
TC (mmol/L)	4.82 ± 0.90	4.27 ± 1.16	<.001	4.62 ± 1.55	4.21 ± 1.10	4.13 ± 1.18	4.59 ± 1.06	4.23 ± 1.12	4.20 ± 1.10	.233
TG (mmol/L)	1.33 ± 1.20	1.88 ± 1.51	<.001	1.60 ± 0.84	1.80 ± 1.30	1.73 ± 1.19	2.21 ± 1.71	2.21 ± 1.91	1.71 ± 1.43	.122
Ca (mmol/L)	2.34 ± 0.11	2.38 ± 0.28	.013	2.21 ± 0.39	2.25 ± 0.15	2.18 ± 0.25	2.31 ± 0.31	2.51 ± 0.21	2.49 ± 0.24	<.001
Adjusted Ca (mmol/L)	2.16 ± 0.14	2.42 ± 0.27	<.001	2.26 ± 0.42	2.28 ± 0.13	2.26 ± 0.22	2.36 ± 0.28	2.54 ± 0.20	2.53 ± 0.22	<.001
Phosphorus (mmol/L)	1.18 ± 0.15	2.08 ± 0.57	<.001	1.63 ± 0.77	1.86 ± 0.55	1.96 ± 0.63	2.15 ± 0.62	2.21 ± 0.39	2.23 ± 0.50	<.001
ALP (U/L)	69.65 (56.03, 82.98)	126.80 (77.38, 371.75)	<.001	68.95 (60.00, 92.40)	70.00 (52.90, 88.80)	75.60 (62.05, 103.70)	108.30 (71.50, 160.85)	161.05 (114.55, 300.53)	462.00 (303.00, 791.00)	<.001
Ln (ALP)	4.24 ± 0.29	5.15 ± 0.99	<.001	4.30 ± 0.33	4.24 ± 0.34	4.38 ± 0.36	4.76 ± 0.68	5.30 ± 0.76	6.11 ± 0.78	<.001
iPTH (pg/mL)	31.72 (22.97, 41.13)	930.40 (160.65, 1792.50)	<.001	24.96 (17.65, 38.74)	93.36 (67.20, 114.70)	207.60 (164.25, 250.95)	608.70 (393.75, 728.55)	1131.50 (945.58, 1285.75)	2344.00 (1875.50, 3241.00)	<.001
Ln (iPTH)	3.40 ± 0.47	6.29 ± 1.56	<.001	3.15 ± 0.64	4.49 ± 0.32	5.33 ± 0.22	6.28 ± 0.33	7.02 ± 0.17	7.82 ± 0.37	<.001
(1-84) PTH (pg/mL)	23.05 (16.81, 33.61)	448.60 (99.62, 850.45)	<.001	21.26 (12.38, 35.90)	64.81 (49.68, 82.35)	124.90 (99.93, 150.05)	302.00 (235.75, 369.30)	549.40 (456.35, 645.57)	1156.00 (865.85, 1523.00)	<.001
Ln [ (1-84) PTH]	3.13 ± 0.48	5.70 ± 1.42	<.001	2.86 ± 1.03	4.15 ± 0.35	4.83 ± 0.35	5.66 ± 0.32	6.30 ± 0.22	7.08 ± 0.43	<.001
(7-84) PTH (pg/mL)	6.29 (3.79, 9.98)	468.20 (54.22, 922.55)	<.001	5.62 (–0.01, 9.99)	24.70 (17.22, 34.34)	72.70 (58.35, 92.75)	261.40 (167.45, 338.30)	589.65 (481.20, 667.10)	1201.00 (959.00, 1738.50)	<.001
Ln [ (7-84)PTH]	1.87 ± 0.88	5.47 ± 1.80	<.001	1.74 ± 0.89	3.14 ± 0.58	4.28 ± 0.45	5.45 ± 0.47	6.33 ± 0.23	7.14 ± 0.43	<.001
(1-84) PTH/iPTH	0.79 ± 0.17	0.58 ± 0.18	<.001	0.85 ± 0.33	0.72 ± 0.10	0.63 ± 0.14	0.55 ± 0.10	0.49 ± 0.07	0.49 ± 0.09	<.001

Data are presented as means ± SD or median (interquartile range) for continuous variables and as n (%) for categorical variables. CKD: chronic kidney disease; PTH: parathyroid hormone; BMI: body mass index; SBP: systolic blood pressure; DBP: diastolic blood pressure; CGN, chronic glomerulonephritis; DN, diabetic nephropathy; HN, hypertensive nephropathy; PKD: polycystic kidney disease; CCB, calcium channel blocker; ACEI/ARB, angiotensin converting enzyme inhibitors/angiotensin receptor blocker; ALP, alkaline phosphatase; Ca, calcium; TC: total cholesterol; TG: triglyceride.

^a^Controls *vs.* CKD5 patients. ^b^Analysis of variance of 6 groups in CKD5 patients.

The CKD5 patients were divided into six groups according to the plasma iPTH levels: iPTH ≤ 50 pg/mL (*n* = 30), 50 < iPTH ≤ 150 pg/mL (*n* = 53), 150 < iPTH ≤ 300 pg/mL (*n* = 41), 300 < iPTH ≤ 800 pg/mL (*n* = 37), 800 < iPTH ≤ 1500 pg/mL (*n* = 80), and iPTH >1500 pg/mL (*n* = 113). With an increase in the circulating iPTH levels, the ALP, (1-84) PTH, and (7-84) PTH levels all increased and the (1-84) PTH/iPTH ratio gradually decreased (Table 1).

### The HRV characteristics in the different subgroups and the independent influencing factors

**Figure 1.**
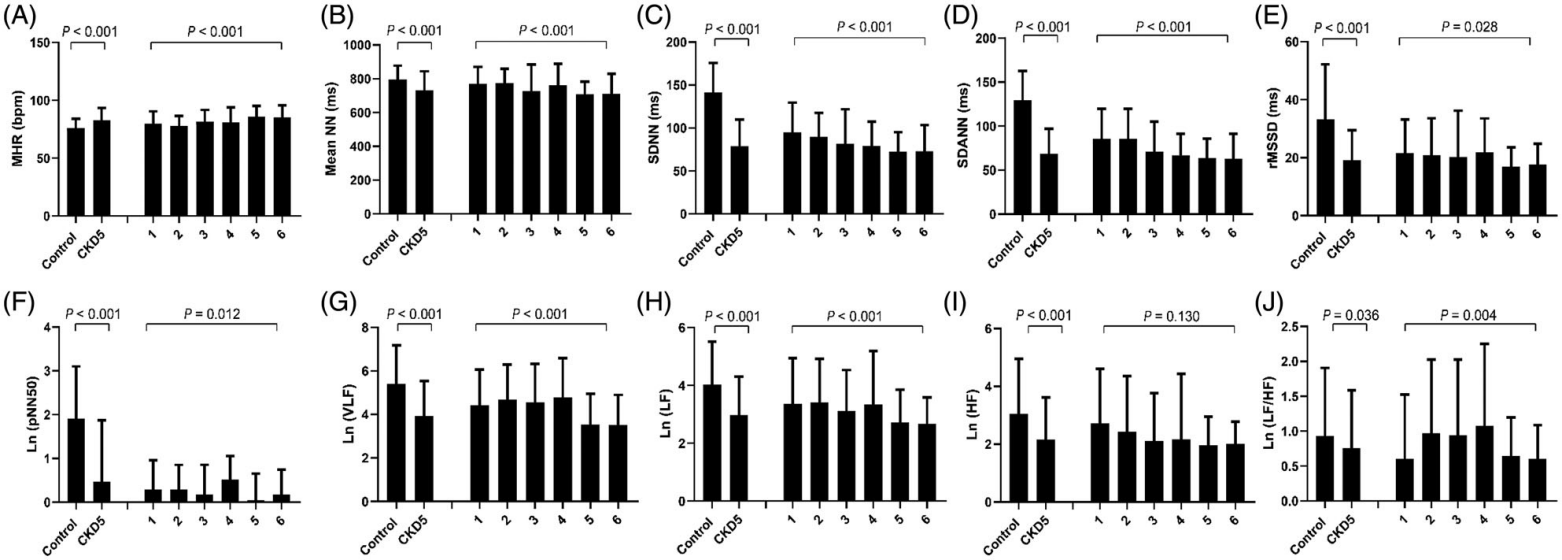
Baseline HRV indices in CKD5 patients and healthy controls. CKD5 patients were divided into 6 groups according to plasma iPTH levels: 1: iPTH ≤ 50pg/mL (*n* = 30); 2: 50 < iPTH ≤ 150pg/mL (*n* = 53); 3: 150 < iPTH ≤ 300pg/mL (*n* = 41); 4: 300 < iPTH ≤ 800pg/mL (*n* = 37); 5: 800 < iPTH ≤ 1500pg/mL (*n* = 80); 6: iPTH >1500pg/mL (*n* = 113). MHR: mean 24-h heart rate; Mean NN: mean normal-to-normal R-R intervals; SDNN: standard deviation of the normal-to-normal R-R intervals; SDANN: standard deviation of 5-min average of normal R-R intervals; rMSSD: root-mean square of differences between adjacent normal R-R interval; pNN50: percentage of adjacent NN intervals differing by more than 50 milliseconds; VLF: very-low frequency; LF: low-frequency; HF: high-frequency.

In the stage 5 CKD patients, the MHR was significantly higher than in the controls (*p* < .001), while the time domain indices including the mean NN, SDNN, SDANN, rMSSD, pNN50, and frequency domain indices (VLF, LF, and HF) were significantly lower than controls. When the plasma iPTH levels increased, the MHR rose in each subgroup, especially when the iPTH levels were greater than 800 pg/mL (*p* < .001). Furthermore, the time domain indices (mean NN, SDNN, SDANN, rMSSD, and pNN50) and frequency domain indices (lnVLF, lnLF, and lnHF) were significantly decreased when the plasma iPTH was greater than 800 pg/mL in the CKD5 patients (Figure 1).

To eliminate the influence of potential confounding factors, a multiple stepwise regression model was established. By using all of the HRV indices as dependent variables, associations were observed between the HRV and the bone metabolism markers. In the CKD patients, the circulating iPTH levels were independently and inversely correlated with the mean NN, Ln (VLF), Ln (LF), and Ln (HF), while the (1-84) PTH was negatively related with the SDNN and SDANN. There were no correlations between the baseline (7-84) PTH levels and the HRV indices. Additionally, the serum adjusted calcium levels were correlated with the MHR, the mean NN, the rMSSD, and pNN50. The hemoglobin, blood albumin, glucose, creatinine levels and the dialysis vintage were independently related with the HRV indices (Table 2).

**Table 2.** Influencing factors of HRV in CKD5 patients analyzed by multiple stepwise regression.

Dependent Variable	Independent Variable	Unstandardized Coefficient	Standardized Coefficient	95% CI	*p* Value	Adjusted R^2^
MHR (bpm)	BMI	0.41	0.13	(0.09, 0.74)	.013	0.13
	Creatinine	0.00	0.11	(0.00, 0.01)	.032	
	Ln (ALP)	2.21	0.21	(1.08, 3.34)	<.001	
	Adjusted Ca	7.99	0.21	(3.82, 12.16)	<.001	
Mean NN (ms)	Ln (iPTH)	–12.20	–0.16	(–21.20, −3.20)	.008	0.05
	Adjusted Ca	–51.04	–0.12	(–100.36, −1.72)	.043	
SDNN (ms)	Hb	0.25	0.17	(0.09, 0.41)	.003	0.14
	Glu	–2.50	–0.15	(–4.40, −0.60)	.010	
	Alb	0.99	0.15	(0.27, 1.70)	.007	
	Ln [(1-84) PTH]	–6.98	–0.31	(–9.44, −4.52)	<.001	
SDANN (ms)	Hb	0.22	0.16	(0.07, 0.37)	.005	0.16
	Glu	–2.44	–0.15	(–4.20, −0.68)	.007	
	Alb	1.07	0.18	(0.42, 1.72)	.001	
	Ln [(1-84) PTH]	–7.01	–0.34	(–9.28, −4.73)	<.001	
rMSSD (ms)	TG	–0.94	–0.13	(–1.69, −0.18)	.015	0.03
	Adjusted Ca	–5.64	–0.15	(–9.74, −1.54)	.007	
pNN50 (%)	Age	0.07	0.14	(0.01, 0.12)	.015	0.03
	Adjusted Ca	–2.47	–0.11	(–4.82, −0.11)	.040	
Ln (VLF)	Age	–0.53	–0.17	(–0.87, −0.19)	.002	0.20
	Dialysis vintage	0.00	–0.14	(–0.01, 0.00)	.024	
	Hb	–0.02	–0.22	(–0.02, −0.01)	<.001	
	Glu	–0.11	–0.13	(–0.21, −0.02)	.017	
	Creatinine	0.00	–0.11	(0.00, 0.00)	.050	
	Ln (iPTH)	–0.32	–0.31	(–0.45, −0.20)	<.001	
Ln (LF)	Sex	–0.43	–0.16	(–0.72, −0.14)	.003	0.08
	Age	–0.01	–0.11	(–0.03, −0.00)	.041	
	Ln (iPTH)	–0.21	–0.24	(–0.30, −0.11)	<.001	
Ln (HF)	Sex	–0.36	–0.12	(–0.68, −0.03)	.031	0.04
	Dialysis vintage	0.00	0.14	(0.00, 0.01)	.027	
	Ln (iPTH)	–0.20	–0.20	(–0.32, −0.07)	.002	
Ln (LF/HF)	Dialysis vintage	–0.00	–0.21	(–0.01, −0.00)	<.001	0.04

Multiple stepwise regression models with adjustment for age, sex, BMI, SBP, dialysis vintage and Hb, Glu, Alb, Ln (ALP), TC, TG, Creatinine, adjusted Ca, P, Ln (iPTH), Ln [(1-84) PTH], Ln [(7-84) PTH] and (1-84) PTH/iPTH. CKD: chronic kidney disease; PTH: parathyroid hormone; BMI: body mass index; SBP: systolic blood pressure; Hb: hemoglobin; Glu: glucose; ALP, alkaline phosphatase; TC: total cholesterol; TG: triglyceride; Ca, calcium; P: phosphorus; MHR: mean 24-h heart rate; Mean NN: mean normal-to-normal R-R intervals; SDNN: standard deviation of the normal-to-normal R-R intervals; SDANN: standard deviation of 5-min average of normal R-R intervals; rMSSD: root-mean square of differences between adjacent normal R-R interval; pNN50: percentage of adjacent NN intervals differing by more than 50 milliseconds; VLF: very-low frequency; LF: low-frequency; HF: high-frequency; CI: confidence interval.

### The effects of the PTX on the plasma PTH fragments and the HRV indices

**Figure 2.**
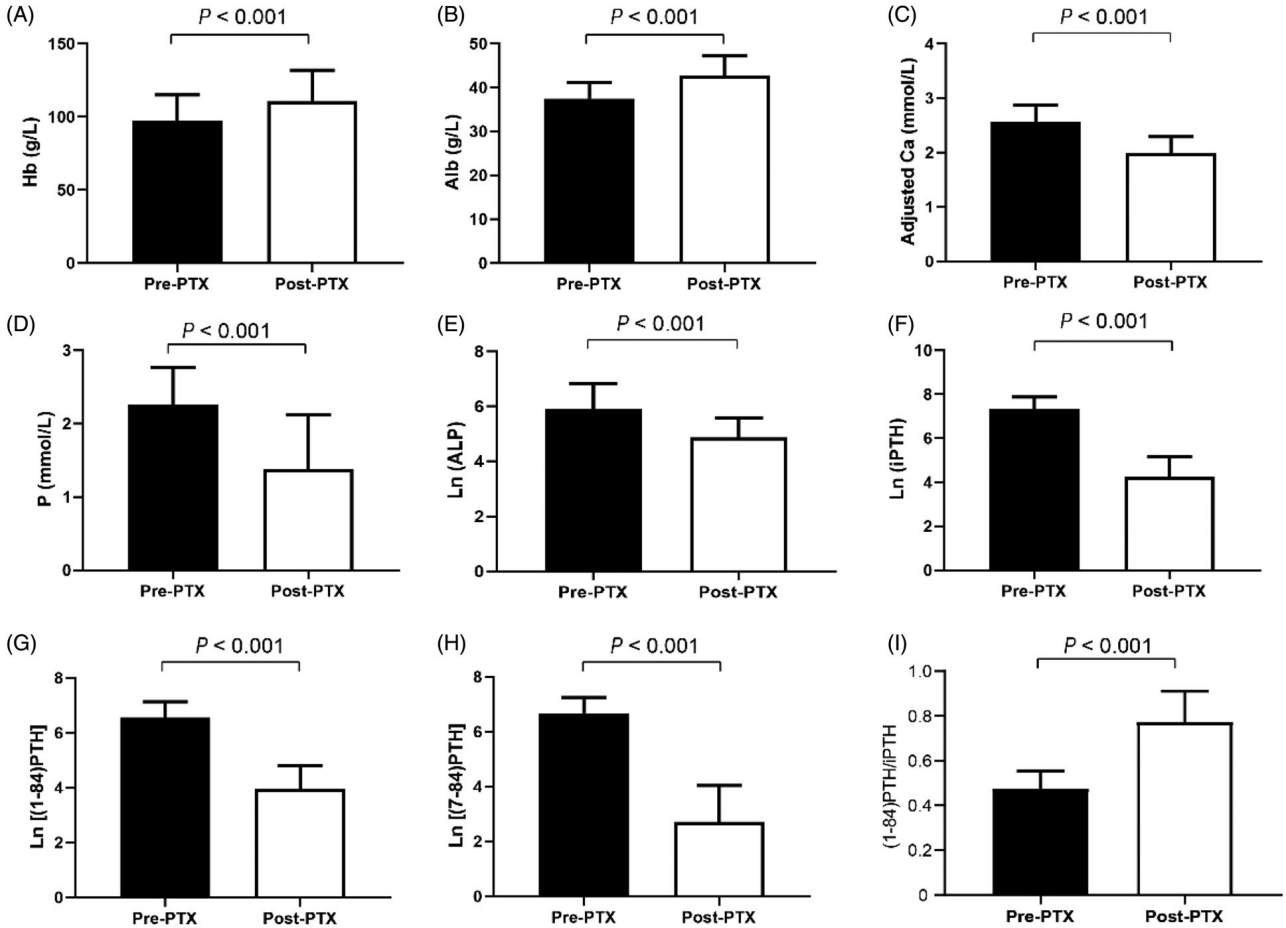
Comparisons of laboratory indices in PTX patients. PTX: parathyroidectomy; Hb: hemoglobin; Alb: albumin; Ca: calcium; P: phosphorus; ALP: alkaline phosphatase; iPTH: intact parathyroid hormone.

Thirty PTX patients were followed up with a median interval of 6.5 months. As shown in Figure 2, anemia, hypoalbuminemia, and bone metabolism disorders were improved after PTX. The levels of phosphorus, adjusted calcium, ALP, iPTH, (1-84) PTH, and (7-84) PTH decreased, while the (1-84) PTH/iPTH ratio increased significantly in the SHPT patients after PTX.

In addition, the HRV indices were significantly improved in the CKD patients after PTX, as shown in Table 3. The MHR decreased, while the time domain and frequency domain indices increased significantly, except for Ln (LF/HF).

**Table 3.** Pre- and post-PTX HRV indices in CKD5 patients.

Variable	Pre-PTX	Post-PTX	*p* Value
MHR (bpm)	88.97 ± 9.70	78.60 ± 9.63	<.001
*Time domain*			
Mean NN (ms)	684.33 ± 74.87	774.50 ± 100.70	<.001
SDNN (ms)	66.40 ± 18.64	100.27 ± 22.84	<.001
SDANN (ms)	56.20 ± 16.41	86.80 ± 26.15	<.001
rMSSD (ms)	17.63 ± 6.68	23.23 ± 10.19	.010
pNN50 (%)	2.37 ± 2.78	5.54 ± 6.25	.015
*Frequency domain*			
Ln (VLF)	5.40 ± 0.93	5.90 ± 0.85	.004
Ln (LF)	3.98 ± 1.35	4.70 ± 1.2242	.014
Ln (HF)	2.80 ± 1.64	3.75 ± 1.4944	.020
Ln (LF/HF)	0.96 ± 0.79	0.62 ± 0.69	.089

Data are presented as means ± SD or median (interquartile range) for continuous variables. HRV: heart rate variability; PTX: parathyroidectomy; CKD: chronic kidney disease; MHR: mean 24-h heart rate; Mean NN: mean normal-to-normal R-R intervals; SDNN: standard deviation of the normal-to-normal R-R intervals; SDANN: standard deviation of 5-min average of normal R-R intervals; rMSSD: root-mean square of differences between adjacent normal R-R interval; pNN50: percentage of adjacent NN intervals differing by more than 50 milliseconds; VLF: very-low frequency; LF: low-frequency; HF: high-frequency.

### Correlations between changes in the HRV indices and the plasma PTH fragments levels after PTX

**Figure 3.**
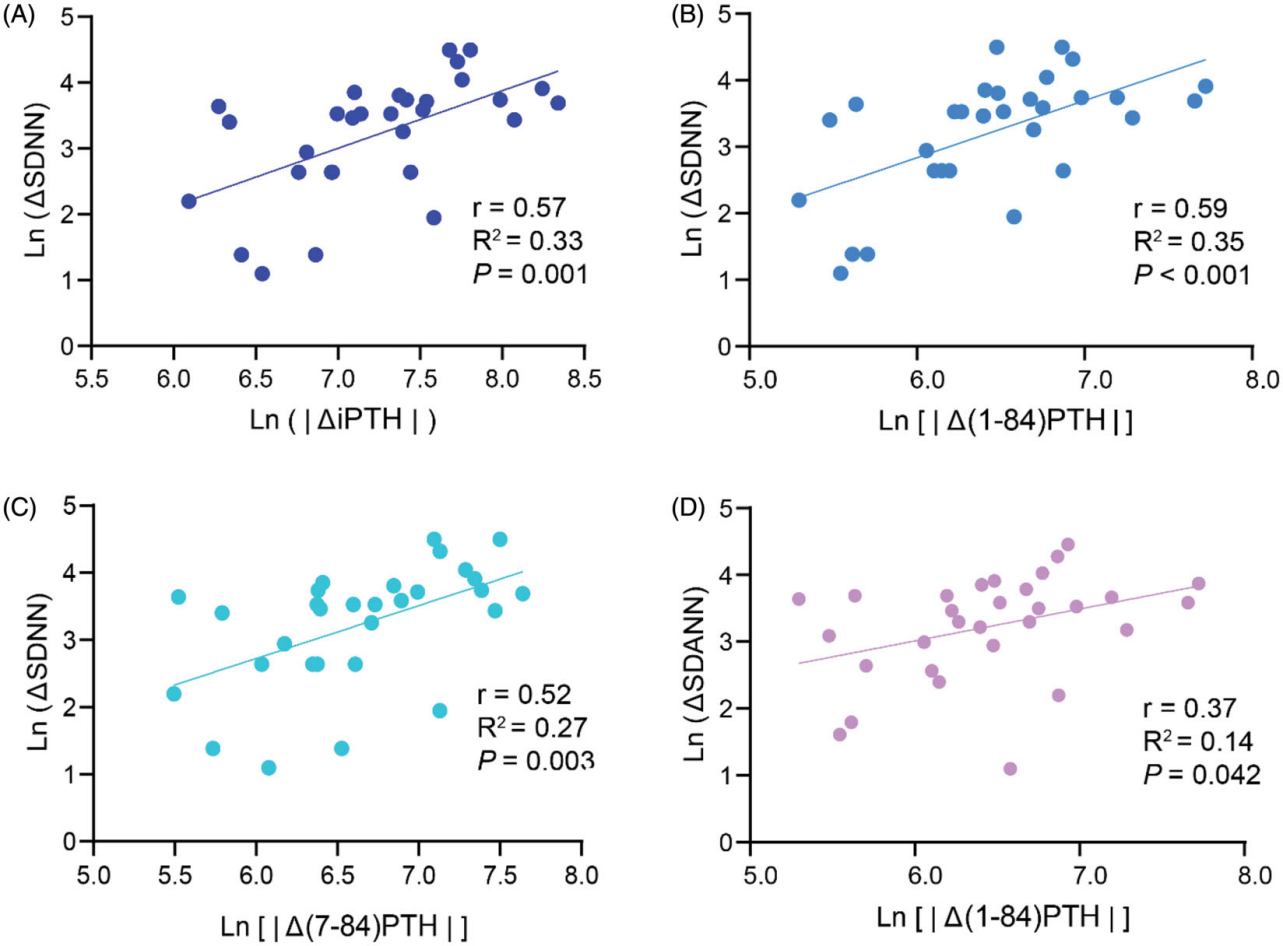
Correlations between the changes of plasma PTH fragments levels and HRV indices in PTX patients before and after operation. Linear correlations between SDNN and Ln (iPTH) (A), Ln[(1-84) PTH] (B) and Ln [(7-84) PTH] (C) in post-PTX patients. Linear correlation between SDANN and Ln[(1-84) PTH] (D) in post-PTX patients (*p* = .042). PTX: parathyroidectomy; iPTH: intact parathyroid hormone. Δ: subtracting the values before PTX from the values of post-PTX; |Δ|: absolute value.

Correlations between the longitudinal improvements of the HRV parameters and the plasma PTH fragments levels are displayed in Figure 3. With a median follow up time of 6.50 months after PTX (*n* = 30), the improved SDNN and SDANN were related with decreased plasma (1-84) PTH levels. Furthermore, the improved SDNN was related with the decreased plasma (7-84) PTH levels.

## Discussion

Chronic kidney disease-mineral and bone disorder (CKD-MBD) is associated with high morbidity and mortality [20]. Although great breakthroughs have been made in the treatment of SHPT, ESKD patients often display significant increases in PTH levels. PTH is synthesized and secreted by the parathyroids, which is a reliable marker of parathyroid function and plays an important role in mineral metabolism [21]. Accurate detection of the circulating PTH levels is essential for the clinical management of CKD-MBD.

The bioactive domain of PTH is located in the N-terminal 1-34 amino acids [14]. (1-84) PTH activates a number of different signaling pathways by binding to a single receptor in the bones and kidneys. In the kidneys, it increases calcium reabsorption, inhibits phosphate reabsorption, and stimulates the conversion of 25-hydroxyvitamin D3 to 1,25-dihydroxyvitamin D3, which promotes intestinal calcium and phosphate absorption [22]. PTH can stimulate bone degradation, which leads to an increase in calcium and phosphate release [22]. Furthermore, PTH induces myocardial dysfunction and cardiac hypertrophy [23,24].

Now the most widely used method for measuring the PTH levels is the second generation iPTH assay, detecting both the full-length (1-84) PTH and long C-PTH fragments, primarily (7-84) PTH. The third generation PTH assays are specific for (1-84) PTH. The importance of (7-84) PTH in the composition of iPTH increased with a decrease in the eGFR, being 21% in the healthy individuals, 32% in the progressive renal failure patients with GFRs < 30 mL/min/1.73m^2^, and 50% in the HD patients [25]. It has been reported that the (1-84) PTH/iPTH ratio was a better clinical indicator than iPTH or (1-84) PTH for evaluating bone turnover in CKD patients, predicting the effectiveness of PTX, and estimating the fatality rate among hemodialysis patients [26]. In the CKD5 patients, the (1-84) PTH/iPTH ratio was between 0.4 and 0.7 [27,28], which was similar to the results of this study. The (7-84) PTH accounted for 21% in the healthy controls and 43% in the stage 5 CKD patients. Thus, the second generation iPTH assay may overestimate the severity of hyperparathyroidism in CKD patients, especially in severe SHPT individuals.

It has been revealed that (7-84) PTH can antagonize the biological effects of (1-84) PTH on the bones and kidneys through the receptor (C-PTHR) binding to the carboxyl terminal region of PTH. Slatopolsky et al. found that in normal parathyroidectomized rats, when (1-84) PTH and (7-84) PTH were given simultaneously in a 1:1 ratio, the calcemic response to (1-84) PTH was reduced by 94%, and the phosphaturic response to (7-84) PTH was decreased by 50.2% [29]. It has been shown that (7-84) PTH can reduce PTH1 receptor expression in bone and kidney derived cell lines through mechanisms involving receptor internalization [30]. However, the effects of (1-84) PTH and (7-84) PTH on the CVD remain unclear.

Autonomic nerve dysfunction, especially sympathetic hyperfunction can induce arrhythmia, which is an important factor in the sudden death and abnormal cardiovascular rhythm in dialysis patients [31]. The HRV is an effective method to evaluate cardiac autonomic nervous function and has an independent prognostic value in chronic hemodialysis patients with increased risks for all-cause and sudden death [32]. The decreased HRV indices in ESKD patients reflect the impairment of the autonomic nervous regulation on the cardiovascular system, and is an independent predictor of cardiac death [8].

In this study, the time and frequency domain HRV indices in CKD5 patients were significantly lower than in the controls. The SDNN reflects the balance of the overall sympathetic and parasympathetic functions. Oikawa et al. reported that an SDNN <75 ms was closely related with all-cause and cardiovascular death with a follow-up of 2110 ± 903 days in 383 maintenance dialysis patients [33]. Buonacera et al. reported that a SDNN< 96 ms was an independent risk factor for cardiovascular death in asymptomatic elderly hypertensive patients [34]. In this study, it was shown that the average value of the SDNN in CKD5 patients was 78.82 ms, while in severe SHPT patients it was further decreased to 72.93 ms. This result suggested that patients with CKD and severe SHPT are at high risk of CVD and death.

PTH has a variety of cardiovascular effects, including the induction of cardiomyocytes apoptosis and promoting myocardial interstitial fibrosis, leading to endothelial dysfunction that is independent of blood phosphorus to cause vascular calcification and is closely related to left ventricular hypertrophy [35,36]. PTH not only affects the structure and function of the heart, but also is related to the disorder of the HRV indices and the autonomic nerve function in CKD patients. An increase in the blood iPTH levels may aggravate the damage to the autonomic nervous system [11,37,38]. The aim of this study was to observe the relationships between iPTH, (1-84) PTH, (7-84) PTH, and the HRV indices in CKD5 patients. A multiple stepwise regression analysis revealed that circulating iPTH levels were independently and inversely correlated with the mean NN, Ln (VLF), Ln (LF), and Ln (HF), while (1-84) PTH was negatively correlated with the SDNN and SDANN. However, there were no correlations between the baseline plasma (7-84) PTH levels and the HRV indices.

Our multiple stepwise regression analysis also showed serum adjusted calcium levels were negatively correlated with the Mean NN, rMSSD and pNN50, while positively correlated with MHR. However, there were no references about the associations of circulating adjusted calcium levels and HRV. In a cross-sectional study with 116 adult women, circulating magnesium (Mg) levels were negatively correlated with MHR and positively correlated with SDNN [39]. Another study demonstrated that plasma Mg levels showed a positive correlation with pNN50 and HF, indicating that Mg might enhance the parasympathetic activity resulting in a cardio-protection in hemodialysis patients [40].

Low iPTH levels have been reported to have adverse outcomes for CKD patients [41–43], which reflects the poor nutritional status related to protein-energy malnutrition and chronic inflammation in dialysis patients. It can also accelerate the occurrence of atherosclerotic cardiovascular disease, be related to adynamic osteopathy caused by overtreatment, and promote vascular calcification [44–46]. In this study, it was found that compared with the controls, the time domain indices (including SDNN, SDANN, rMSSD, and pNN50) and the frequency domain indices (including VLF and LF) were significantly decreased in the subgroup whose plasma iPTH levels were under 50 pg/ml (results not shown). The effects of low plasma iPTH levels on cardiovascular outcomes and mortality in CKD5 patients require further investigation.

Successful PTX can reverse the disorders of bone mineral metabolism and HRV indices and reduce cardiovascular and all-cause mortality in CKD5 patients [6,12]. We have demonstrated that in PTX patients, improved HRV indices were correlated with decreased blood levels of bone biomarkers [iPTH, bone-specific alkaline phosphatase (BAP), tartrate-resistant acid phosphatase 5 b (TRACP-5b), fibroblast growth factor 23 (FGF23)] [38]. In this study, patients with PTX were followed up over three months after the surgery (median follow-up time: 6.50 months). Consistent with the previous findings [13], elevated circulating adjusted calcium, iPTH, (1-84) PTH, (7-84) PTH levels, and a decreased (1-84) PTH/iPTH ratio was alleviated after PTX in severe SHPT patients. This data showed that an improved SDNN was related with decreased (1-84) PTH and (7-84) PTH levels, while improved SDANN was related with decreased (1-84) PTH levels. No antagonistic effects from (1-84) PTH and (7-84) PTH on the HRV were found in the CKD5 patients. Chou et al. [47] revealed that in SHPT patients with PTX, calcification scores of abdominal aorta (CSAA) were improved after 1 year. Since the median follow-up time of 30 PTX patients in this study was 6.5 months, we speculated that the improvements of HRV indices in PTX patients were mainly attributed to autonomic nervous regulation, rather than ameliorations of vascular calcification. However, the relevant mechanisms remain unclear.

There do exist some limitations in this study. First, direct cause-and-effects relationships between the PTH fragments and the HRV in the CKD5 patients could not be confirmed. Second, until now, the plasma (7-84) PTH levels could not be examined directly and instead of they were calculated by subtracting the plasma (1-84) PTH levels from the plasma iPTH levels, which actually contain various long C-PTH fragments. Third, we didn’t collect the baseline and post-operative magnesium levels in CKD5 patients and couldn’t analyze the associations of HRV parameters and circulating magnesium levels. Finally, less PTX patients were followed up and might be related with an unmeasurable bias. A larger sample size and more basic research regarding the different PTH fragments and CVD need to be investigated.
